# Size-dependent phase transition in methylammonium lead iodide perovskite microplate crystals

**DOI:** 10.1038/ncomms11330

**Published:** 2016-04-21

**Authors:** Dehui Li, Gongming Wang, Hung-Chieh Cheng, Chih-Yen Chen, Hao Wu, Yuan Liu, Yu Huang, Xiangfeng Duan

**Affiliations:** 1 Department of Chemistry and Biochemistry, University of California, 607 Charles E. Young Drive East, Los Angeles, California 90095, USA; 2 California Nanosystems Institute, University of California, Los Angeles, California 90095, USA; 3 Department of Materials Science and Engineering, University of California, Los Angeles, California 90095, USA

## Abstract

Methylammonium lead iodide perovskite has attracted considerable recent interest for solution processable solar cells and other optoelectronic applications. The orthorhombic-to-tetragonal phase transition in perovskite can significantly alter its optical, electrical properties and impact the corresponding applications. Here, we report a systematic investigation of the size-dependent orthorhombic-to-tetragonal phase transition using a combined temperature-dependent optical, electrical transport and transmission electron microscopy study. Our studies of individual perovskite microplates with variable thicknesses demonstrate that the phase transition temperature decreases with reducing microplate thickness. The sudden decrease of mobility around phase transition temperature and the presence of hysteresis loops in the temperature-dependent mobility confirm that the orthorhombic-to-tetragonal phase transition is a first-order phase transition. Our findings offer significant fundamental insight on the temperature- and size-dependent structural, optical and charge transport properties of perovskite materials, and can greatly impact future exploration of novel electronic and optoelectronic devices from these materials.

## Abstract (web summary)

The orthorhombic-to-tetragonal phase transition in perovskite can alter its optical and electronic properties. Here, Li *et al*. report an investigation of the size dependent orthorhombic-to-tetragonal phase transition and show that the phase transition temperature decreases with reducing microplate thickness.

## Graphical abstract

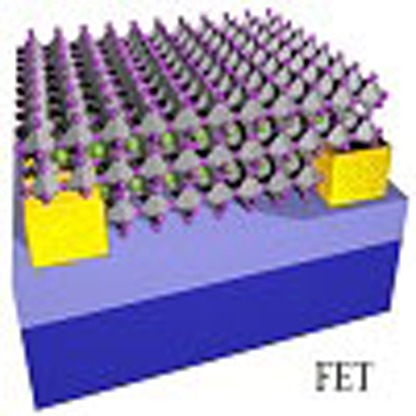

The hybrid organic–inorganic methylammonium lead iodide perovskite (CH_3_NH_3_PbI_3_, denoted as MAPbI_3_) is emerging as one of most promising solution-processable light absorber for solar cells and thus has attracted intensive recent interest^1^,^2^,^3^,^4^,^5^,^6^,^7^,^8^,^9^. With long carrier diffusion length^10^,^11^,^12^ and low non-radiative recombination rate, solution processed perovskite materials have been demonstrated to deliver a certified power-conversion efficiency as high as 20.1% in the past a few years^13^. The excellent optical properties of MAPbI_3_ perovskite enables it to be applied in a wide range of optoelectronic devices such as photodetectors^14^, lasers^15^,^16^,^17^ and light-emitting diodes^18^. Despite the tremendous interest in MAPbI_3_ perovskite, its charge transport properties remain elusive because of the ion motion, which leads to a very large hysteresis and prevents the observation of the intrinsic field-effect mobility at the room temperature^19^,^20^. In addition, it has been proven that the solar cell efficiency strongly depends on the size of cuboids of perovskites^21^. Therefore, it is expected that the size could significantly influence the optical and charge transport properties of MAPbI_3_, yet there is no systematic investigation of size-dependent optical and charge transport properties in perovskite materials.

The structural phase transitions can significantly alter the optical and electronic properties of materials^22^, both of which are essential to understand the underlying photophysics^23^. The temperature-dependent studies such as photoluminescence (PL) spectroscopy^23^, neutron powder diffraction^24^, calorimetric and infrared spectroscopy^25^ have been utilized to investigate the structural phase transitions in bulk MAPbI_3_. The MAPbI_3_ adopts the simple cubic perovskite structure above 330 K, transits to the tetragonal phase at 330 K (refs 26, 27), and further evolves into an orthorhombic phase as the temperature is reduced to 160 K (ref. 27). All those phase transitions have been proven to be of first order^25^. Previous studies have shown that the physical size of a material can be an important variable in determining the phase transition points in addition to pressure, temperature and compositions^28^,^29^,^30^. Therefore, it is important to investigate how the size alters the phase transition points in MAPbI_3_, which in turn affects its optical and electronic properties^31^,^32^. Nevertheless, the size-dependent phase transition in MAPbI_3_ remains elusive. Here we report a systematic investigation of size-dependent structural phase transitions in individual MAPbI_3_ microplate crystals by using temperature-dependent charge transport measurements, PL spectroscopy and transmission electron microscopy and electron diffraction.

## Results

### Temperature-dependent electrical measurement

**Figure 1:**
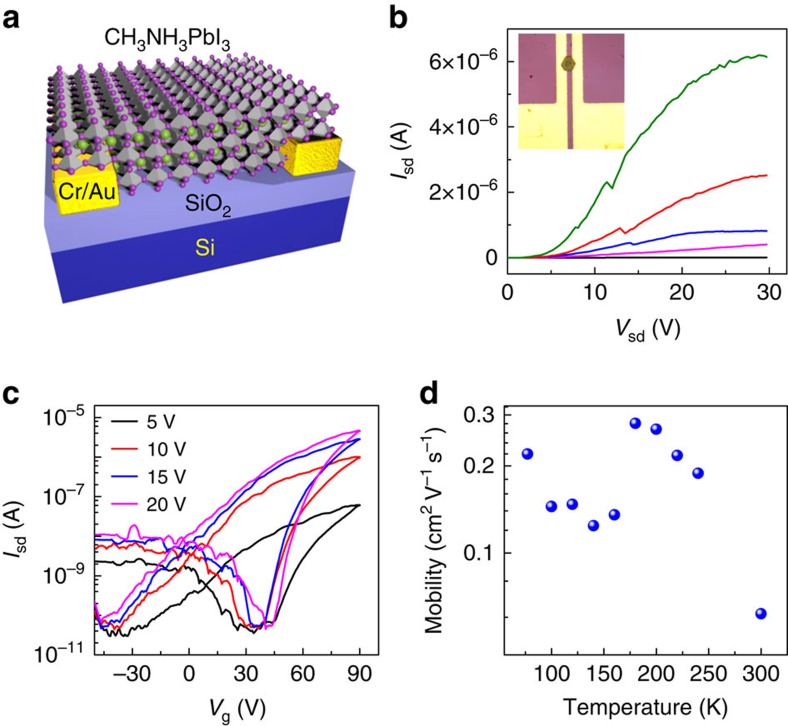
Field-effect transistors based on individual perovskite microplate. (**a**) Schematic of the bottom-gate, bottom-contact halide perovskite microplate field-effect transistor fabricated on a 300-nm SiO_2_/Si substrate with 5 nm Cr/50 nm Au as contact. (**b**,**c**) The output (*V*_g_=0, 20, 40, 60, 80 V; from bottom to top; **b**) and transfer (*V*_sd_=5, 10, 15, 20 V; **c**) characteristics of a field-effect transistor based on a perovskite crystal microplate at 77 K. The inset of **b** shows an optical image of a typical device. The channel length is around 8 μm. (**d**) The temperature-dependent field-effect electron mobility measured with a source-drain voltage of 20 V.

To investigate the fundamental charge transport properties of individual perovskite microplates, we have constructed field-effect transistors (FETs) using the perovskite crystals and measured their transistor characteristics from the room temperature to liquid nitrogen temperature in dark. Figure 1a shows a schematic of a typical FET device configuration we used. The individual perovskite microplates served as the semiconducting channel of FET devices bridging two pre-fabricated Cr/Au electrodes as the source-drain electrodes on 300 nm SiO_2_/Si substrate (as both the gate dielectrics and gate electrode). The inset of Fig. 1b displays an optical image of a typical FET device, where the thickness of the perovskite microplate is around 400 nm. Figure 1b shows a set of typical output characteristics (source-drain current *I*_sd_ versus source-drain voltage *V*_sd_) of a single perovskite microplate FET device under various gate voltages (*V*_g_) at 77 K. The large positive gate voltage induces a higher source-drain current, which indicates an n-type conduction behaviour of perovskite microplate (Fig. 1b). The slight nonlinearity of *I*_sd_*–V*_sd_ curves near zero bias suggests that the contact is not fully optimized. The transfer characteristics exhibit dominant n-type behaviour with a slight p-type conductance at negative gate voltage (Fig. 1c). The maximum on/off ratio is nearly six orders of magnitude, which is better than recently reported perovskite thin-film transistors^19^.

Strong hysteresis is commonly observed in perovskite thin-film transistors, which prevents fully understanding the charge transport properties and exact determination of carrier mobility in such perovskite materials. The origin of the hysteresis has been attributed to ferroelectricity, ion motion within the perovskite material and trapping/de-trapping of charge carriers at the interfaces^19^. However, no conclusive explanation is available to date. In our microplate devices, considerable hysteresis has been observed for all temperatures from 296 to 77 K, which reduces with decreasing temperature. It has been proven that the ion motion in halide perovskite is a thermally activated process^33^ and the ion migration rate exponentially reduces as the temperature decreases. The contribution from ion motion to hysteresis is expected to negligibly small at lower temperatures (for example, 77 K). The presence of hysteresis at 77 K suggests that the ion motion only partly contributes to the hysteresis and other factors such as trap states and surface dipoles may play important roles as well^19^. It is important to note that the hysteresis in our microplate device only increases slightly when the temperature is increased from 77 to 296 K (Supplementary Fig. 1), and the hysteresis at 296 K is considerably smaller than that observed in thin-film perovskite FET devices, where the presence of huge hysteresis prevents the observation of field-effect behaviour above 258 K (ref. 19).

Based on the transfer characteristics, the field-effect carrier mobility can be extracted. The existence of hysteresis in transfer characteristics may lead to systematic errors in mobility determination, with possible underestimation in positive sweeping direction, overestimation in negative sweeping direction and scan rate dependence (Supplementary Fig. 2). To this end, we have determined carrier mobility based on both the positive and negative sweepings. Nevertheless, both the positive and negative sweepings give the exactly same trend of the mobility versus temperature. For the simplicity of discussion, we focus on the mobility values derived from negative sweeping here. The field-effect electron mobility continuously increases with the decreasing temperature from 300 to 180 K, and then shows a sudden decrease when the temperature is reduced from 180 to 160 K (Fig. 1d). Afterwards with further decreasing temperature, the field-effect electron mobility starts to increase again. Qualitatively similar temperature-dependent characteristics have been observed in all devices except that the transition temperature varies with the thickness of microplates, which we will discuss below in detail. As there is a structural phase transition from the tetragonal phase to the orthorhombic phase at 160 K (ref. 23), we attribute this sudden decrease of field-effect mobility to the structural phase transition. The structural phase transition would induce the change of effective mass and dielectric constant^19^,^34^,^35^, both of which could contribute to the change of field-effect mobility^19^,^36^.

The theoretical calculation based on semi-classical Boltzman transport theory predicates that the mobility of orthorhombic phase should be larger than that of tetragonal phase^19^. In contrast, we observed a sudden decrease of the mobility when the MAPbI_3_ transits from the tetragonal phase to the orthorhombic phase. Previous optical studies indicate that there are small inclusions of the tetragonal phase domains within the orthorhombic phase even when the temperature is much lower than the tetragonal phase to orthorhombic phase transition temperature (*T*_t-o_), likely due to the strain imposed by the thermal expansion and change of the in-plane lattice constant during the phase transition^23^. Our PL studies also demonstrate the presence of such small inclusions (see below). It is very likely that such small inclusions introduce more boundaries and thus increases the carrier scattering, which also contribute to the sudden decrease of the mobility upon the phase transition. Although the improvement of field-effect mobility with the decreasing temperature can be attributed to the electron–phonon interaction and ion drift under applied electric field within the tetragonal phase^19^,^37^, the origins of the rapid increase of the field-effect mobility within the orthorhombic phase are much more complicated. Previous studies have shown that the minimum phonon energy related to the methylammonium (MA) cation is estimated to be 15 meV (refs 38, 39). Therefore, the interaction of carriers with phonons associated with MA libration should be quenched below 170 K. It has been shown that the quench of the carrier–phonon interaction related to the MA libration modes led to a weaker temperature dependence of carrier mobility below 198 K in halide perovskite thin-film transistors^19^, indicating the strong interaction between the carriers and MA libration modes. Without the contribution from interaction with MA libration modes <170 K, the increase rate of the mobility with decreasing temperature should be slowed if only carrier–phonon interaction contributes to the decrease of the mobility after the phase transition. On the contrary, we observed a different picture: a more rapid increase with decreasing temperature (Fig. 1d). Therefore, we suggest that in addition to the carrier–phonon scattering, the decrease of the small inclusions of tetragonal phase domains and the reduction of the grain boundaries might partly contribute to the rapid increase of mobility with the decreasing temperature. The inclusions of tetragonal phase near the transition point within the orthorhombic phase are also confirmed by our temperature-dependent selected area electron diffraction (SAED) studies (see below).

It should be noted that the electron field-effect mobility we extracted here is smaller than those measured by THz spectroscopy in perovskite films (∼8 cm^2^ V^-1^ s^-1^ at room temperature)^40^, Hall measurement in perovskite single crystals (∼66 cm^2^ V^-1^ s^-1^)^27^ and electrical measurement in the space-charge-limited current regime or time-of-flight measurement in perovskite single crystals (∼ 2.5–25 cm^2^ V^-1^ s^-1^)^10^,^11^, but compares favourably with the field-effect mobility reported in perovskite thin-film transistors (∼0.1 cm^2^ V^-1^ s^-1^)^19^. Time-resolved THz spectroscopy probes short-time dynamics up to a few nanoseconds and thus measures local carrier transport phenomena, whereas electrical measurements focus long-time (μs) or longer conduction processes occurring over several micrometres length-scale of a device^41^,^42^. Electrical measurements are therefore more sensitive to grain size, boundaries and interfacial effects because of the carrier transport over the device dimension. Thus, it is not surprising that the mobility measured by THz spectroscopy is larger than that measured by electrical measurements. Although Hall measurements, time-of-flight technique and space-charge-limited current method directly measure intrinsic charge transport, the field-effect mobility is extremely sensitive to the dielectric/semiconductor interfaces as well as the source, drain contact resistance^43^. Such extrinsic factors in field-effect measurement can often lead to an underestimation of the carrier mobility in FETs.

### Thickness-dependent phase transition

**Figure 2:**
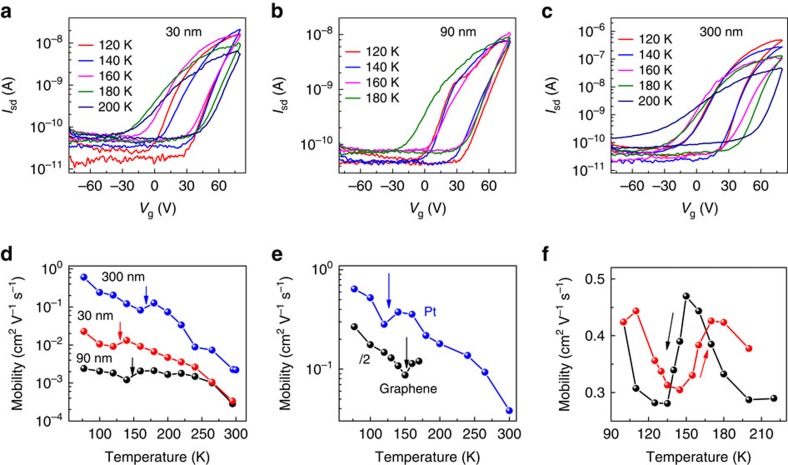
Size-dependent transition of the orthorhombic phase to tetragonal phase. (**a**–**c**) The temperature-dependent transfer characteristics of field-effect transistors made of 30 nm (**a**), 90 nm (**b**) and 300 nm (**c**) thick individual perovskite crystal microplates. The applied source-drain voltage is 20 V and the channel length is 8 μm. (**d**) The temperature-dependent field-effect electron mobility for three different thickness devices extracted from **a**–**c**. The arrows indicate the temperature where the phase transition occurs. The mobility is measured for the heating cycle. (**e**) The temperature-dependent field-effect electron mobility for devices with Pt contact and graphene contact. The thickness of the microplates is around 35 nm and the channel length is 40 μm for Pt contact. For graphene contact, the thickness of the microplates is around 120 nm and the channel length is around 15 μm. The mobility is measured during the heating cycle. (**f**) The temperature-dependent field-effect mobility of a perovskite microplate device (5 nm Cr/50 nm Au as contact) with a thickness of around 200 nm measured for both heating and cooling cycle under a source-drain voltage of 20 V. The channel length of the device is 40 μm.

To investigate how the thickness of the microplates influences the field-effect mobility, we systematically carried out the temperature-dependent transport measurement with different microplate thickness, with the transfer curves (120–200 K) of three representative devices shown in Fig. 2a–c. All three devices exhibit dominant n-type behaviour regardless of the thickness. The field-effect electron mobility extracted from the transfer curves shows a common trend with the temperature for devices with different thickness: as the temperature decreases, the electron mobility first increases, suddenly decreases at the structural phase transition point and increases again with further reducing temperature (Fig. 2d). It is noted that the structural phase transition temperature *T*_t-o_ strongly depends on the thickness of the perovskite microplates: the thicker the microplates are, the higher the structural phase transition temperature *T*_t-o_ is. For the 30-nm-thick microplate, the structural phase transition temperature *T*_t-o_ falls around 130 K, which increases to ∼150 K for the 90 nm microplate, and to ∼170 K for the 400-nm-thick microplate. Furthermore, we have carried out temperature-dependent transport measurement using different metal contact including Pt and graphene (Fig. 2e), on a hexagonal boron nitride (hBN) substrate (Supplementary Fig. 3 and Supplementary Note 1) and in a device with very long channel length (40 μm; Supplementary Fig. 4). It is found that the structural phase transition occurs with phase transition temperature *T*_t-o_ relying only on the thickness of perovskite microplates regardless of the contact materials, substrate and channel length, indicating that the structural phase transition is an intrinsic property of the perovskite microplates.

To precisely locate the tetragonal-to-orthorhombic phase transition point, we scan the temperature range with a higher resolution around the phase transition point for a 200-nm-thick microplate device (Fig. 2f). Similar to the hysteretic behaviour observed in the optical density of perovskite thin films^26^ and dielectric and resistance measurement of MAPbI_3_ crystals^27^,^35^, an apparent hysteresis is observed with a temperature span of 15 K between the cooling cycle and heating cycle. The sharp decrease of the mobility near the phase transition temperature and the presence of the broad hysteresis confirm that the tetragonal phase to the orthorhombic phase transition is a first-order solid–solid phase transition^29^,^44^,^45^.

### Temperature-dependent SAED studies

**Figure 3:**
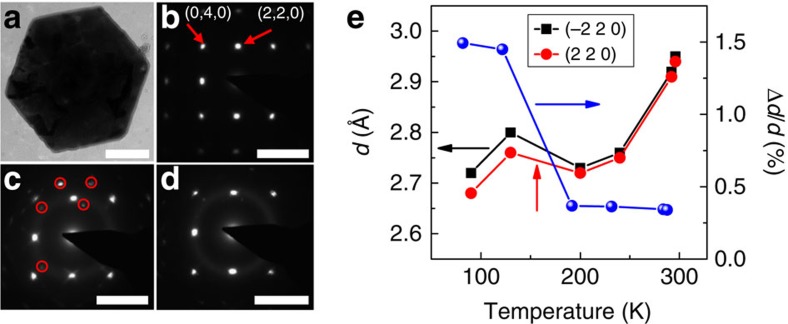
Temperature-dependent selected area electron diffraction (SAED) patterns. (**a**) Low-resolution TEM image of a perovskite microplate. The scale bar is 1 μm. (**b**–**d**) The SAED patterns of the microplate at 296 K (**b**), 90 K (**c**) and 200 K (**d**) along [001] zone axis. The scale bar is 5 nm^−1^. The red circles in **c** indicate the dispersive distributed spots. (**e**) Lattice spacings of (−2 2 0) plane (black squares) and (2 2 0) plane (red squares) of a perovskite microplate. The percentage of lattice spacing difference between those two planes (defined as (*d*(−2 2 0)−*d*(2 2 0))/*d*(2 2 0)) is displayed as well (right axis). The increasing difference between these two lattice spacings with the reducing temperature indicates the transitions from a tetragonal phase to orthorhombic phase.

The tetragonal-to-orthorhombic phase transition can be directly confirmed by the temperature-dependent SAED studies. Transmission electron microscopy (TEM) image shows that the converted perovskite microplates we used to acquire the SAED patterns largely retain hexagonal shape similar with the PbI_2_ microplate before conversion (Fig. 3a). The SAED pattern at room temperature (Fig. 3b) shows a single set of fourfold symmetric diffraction spots that can be indexed to the tetragonal structure of the perovskite crystals along [001] zone axis. Both first-order and second-order diffraction spots can be clearly distinguished, indicating excellent crystalline quality of the perovskite microplate. Decreasing the temperature to 90 K, the SAED pattern shows a set of fourfold symmetric diffraction spots and a few dispersedly distributed spots indicated by red circles (Fig. 3c). Although the fourfold symmetric diffraction spots can be indexed to the first-order diffraction of the orthorhombic structure along [001] zone axis, the dispersedly distributed spots likely belong to a different set of diffraction patterns. Increasing the temperature to 200 K again, the microplate completely transits from orthorhombic phase back to tetragonal phase. The SAED pattern shows similar features as those obtained at room temperature initially, with the dispersedly distributed spots disappeared. The fourfold symmetric spots can be indexed to the tetragonal structure of the perovskite crystals along [001] zone axis (Fig. 3d), implying that those dispersedly distributed spots at 90 K appear only after the tetragonal-to-orthorhombic phase transition completes. Therefore, those dispersedly distributed spots might be originated from the tetragonal phase along different zone axis because of the inclusions of tetragonal domains within orthorhombic phase or from the orthorhombic domains with different crystalline orientation. Nevertheless, based on lattice spacings analysis it is more likely that the inclusions of tetragonal domains contribute to those dispersedly distributed spots. As mentioned above, even when the temperature is much lower than the phase transition temperature, there still are inclusions of tetragonal domains within orthorhombic phase, suggesting that single-crystal tetragonal phase may break into smaller grains of tetragonal phase and orthorhombic phase when the phase transition occurs. Although most of those small grains maintain almost same orientation, some of them significantly deviate, leading to the dispersedly distributed spots. The presence of dispersedly distributed spots also indicates the degradation of the crystalline quality after the tetragonal-to-orthorhombic phase transition.

The tetragonal-to-orthorhombic phase transition can also be identified by carefully analysing the temperature-dependent lattice spacing change. We extracted the lattice spacings for (−2 2 0) plane and (2 2 0) plane of a perovskite microplate from SAED patterns and found that a sudden change of the lattice spacings between 130 and 200 K, indicating the phase change occurs in this temperature regime (Fig. 3e). As the (−2 2 0) plane and (2 2 0) plane are perpendicular to each other, the changing ratio of the lattice spacings between these two planes can be used to identify the crystalline structure as well. Above 200 K, the lattice spacings for those two planes are almost same, indicating that the perovskite microplate has a tetragonal phase. In contrary, there is clear difference between the lattice spacings for those two planes below 200 K, implying the presence of orthorhombic structure (right axis of Fig. 3e). Those results agree with those obtained from transport measurement above.

### Temperature- and thickness-dependent PL studies

**Figure 4:**
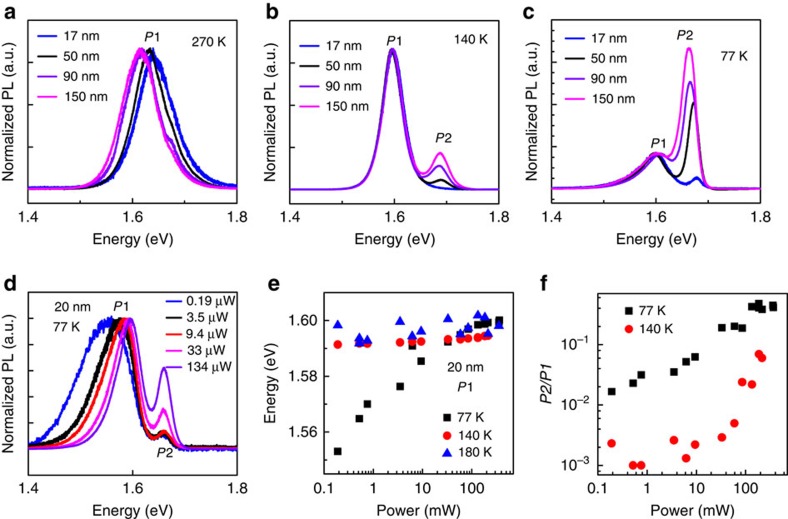
Thickness and excitation power-dependent photoluminescence studies. (**a**–**c**) The photoluminescence spectra for four different thickness halide perovskite microplates at 270 K (**a**), 140 K (**b**) and 77 K (**c**). A 488-nm laser with a power of 3.5 μW was used as the excitation source. All spectra are normalized by the low-energy peak *P*1 in order to easily compare among each other. (**d**) The excitation power-dependent PL spectra for a 20-nm-thick microplate at 77 K. The spectra have been normalized by the low-energy emission peak *P*1. (**e**) The excitation power-dependent emission peak position of the tetragonal phase for the 20-nm perovskite microplate. (**f**) The *P*2/*P*1 ratios extracted from their corresponding PL spectra under different excitation power at 77 K (black squares) and 140 K (red dots).

To further probe the size-dependent tetragonal-to-orthorhombic phase transition, we have also studied temperature-dependent PL. Figure 4a–c displays the PL spectra for four perovskite microplates with various thicknesses at 270, 140 and 77 K. Only one broad emission peak was observed for all four microplates at 270 K and the emission peak shows a blueshift with the reducing thickness (Fig. 4a), which will be discussed in detail below. The PL spectra at 140 K show an extra emission peak at the higher energy for thicker microplates while still exhibits a single peak for the thinner ones (Fig. 4b). Further decreasing the temperature to 77 K, two emission peaks are observed for all microplates but the intensity ratio of the higher energy emission peak (*P*2) to the lower energy emission peak (*P*1) decreases with decreasing microplate thickness (Fig. 4c). The higher energy emission peak can be attributed to the orthorhombic phase, whereas the lower energy emission peak is due to the tetragonal phase domains within the orthorhombic phase^23^, consistent with theoretical calculations that the tetragonal phase has a smaller bandgap than that of the orthorhombic phase^46^. Therefore, the emergence of the two emission peaks signifies the occurrence of phase transition, and our PL studies also suggest that the phase transition temperature *T*_t-o_ is higher for the thicker microplates (Fig. 4b).

### Excitation power-dependent PL spectra

Our excitation power-dependent PL studies also confirms the existence of small tetragonal inclusions below the phase transition temperature *T*_t-o_, which is supported by the fact that both the emission peak position and intensity are extremely sensitive to the excitation power. We have collected excitation power-dependent PL spectra for a 20-nm perovskite microplate in orthorhombic phase (77 K), near orthorhombic-to-tetragonal phase transition point (140 K) and tetragonal phase (180 K), respectively, extracted the emission peak energy and plotted against the excitation power for each peak at 77, 140 and 180 K (Fig. 4d–f and Supplementary Figs 5 and 6). At 77 K, the lower energy emission peak P1 originating from tetragonal phase domains shows an obvious blueshift with the increasing of excitation power, whereas the higher energy peak *P*2 from orthorhombic phase shows little change. It is also noted that the *P*1 emission saturates at high excitation power. As the tetragonal phase has a smaller band gap, the photogenerated carriers prefer to occupy the small tetragonal phase inclusions within the orthorhombic phase. As a result, a large number of carriers are trapped and recombine within those small tetragonal inclusions. As the excitation power increases, the quasi-Fermi levels of the photogenerated carriers move into the conduction band and valence band, resulting in a band filling effect. As the size of the tetragonal inclusions is extremely small, the large blueshift of *P*1 emission peak and saturation of *P*1 intensity can be observed. This sort of blueshift of emission peaks has been commonly observed in quantum wells and other confined heterostructures^47^,^48^. Increasing the temperature to 140 K, similar trend was observed except that the blueshift of *P*1 becomes negligibly small, which is probably due to the increasing size of tetragonal inclusions. With the increasing size of the tetragonal inclusions, the density of states of the tetragonal inclusions increases accordingly, making it more difficult to observe the band filling effect. At 180 K when the orthorhombic-to-tetragonal phase transition has already completed, no noticeable blueshift was observed. It is expected as the excitation power we used is not big enough such that the band filling effect cannot occur in unconfined systems. The excitation power-dependent *P*2/*P*1 ratios also clearly demonstrate the band filling effect (Fig. 4f). At 77 K, the *P*2/*P*1 ratios are very sensitive to the excitation power and show monotonously increases with the increasing excitation power, which indicates the small grains of tetragonal inclusions. At 140 K, the *P*2/*P*1 ratios are always smaller than that at 77 K and only slightly increase with the excitation power, indicating the increasing size of tetragonal inclusions, which renders the band filling effect hard to be observed. Based on the above discussions, we concluded that the presence of the two emission peaks at low temperatures is due to the small inclusions of tetragonal phase domains within the orthorhombic phase.

**Figure 5:**
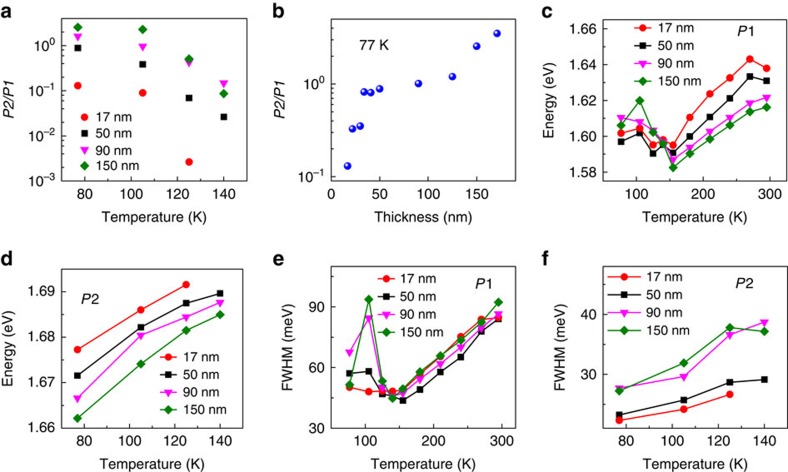
The temperature- and thickness-dependent photoluminescence studies. (**a**) The temperature-dependent *P*2/*P*1 ratios for a 17-, 50-, 90- and 150-nm-thick halide perovskite microplates excited by a 488-nm laser with a power of 3.5 μW. (**b**) The *P*2/*P*1 ratios for halide perovskite microplates with various thicknesses at 77 K. (**c**,**d**) The temperature-dependent emission energy for tetragonal phase (**c**) and orthorhombic phase (**d**). (**e**,**f**) The temperature-dependent full-width at half-maximum (FWHM) for tetragonal phase (**e**) and orthorhombic phase (**f**).

The emission peak energy shows a blueshift with the decreasing microplate thickness both for tetragonal phase above 155 K and the orthorhombic phase below 140 K (Figs 4 and 5, and Supplementary Figs 7 and 8), which has been observed in solution-processed perovskite nanocrystals^49^,^50^. As the thickness of our microplates is much larger than the bulk exciton Bohr radius (2.2 nm) (ref. 51), the quantum confinement effect is unlikely to be the primary factor responsible for this blueshift. Surface effect has been previously proposed to explain such blueshift beyond the quantum confinement regime^52^. In brief, the surface charge-induced depletion electric field near the surfaces or interfaces modifies the confinement potential, leading to a potential well smaller than the actual geometric thickness of the microplates. Our observed blueshift in halide perovskite microplates might be due to the surface effect as well. Nevertheless, the exact underlying mechanism is still unclear and demands further investigation.

### Thickness-dependent phase transition in PL spectra

The *P*2/*P*1 ratios can be used to identify the degree of the phase transition. From *P*2/*P*1 ratios (Fig. 5a,b and Supplementary Fig. 8), we can conclude that the orthorhombic-to-tetragonal phase transition occurs at a lower transition temperature *T*_t-o_ in the thinner microplates, which is consistent with the conclusion obtained from the charge transport measurement. The temperature-dependent PL spectra indicates that the portion of tetragonal inclusions within the orthorhombic phase decreases with the decreasing temperature for all four different thickness microplates, which is supported by the increases of the *P*2/*P*1 intensity ratio with decreasing temperature (Fig. 5a and Supplementary Fig. 8). The transition temperature *T*_t-o_ decreases with the decreasing thickness: *T*_t-o_ <140 K for the thickness smaller than 40 nm, and between 140 and 150 K for the thickness around 40–200 nm. Within the respective tetragonal phase (155–290 K) and orthorhombic phase (77–140 K), the emission peak shows a redshift and the full-width at half-maximum (FWHM) narrows as the temperature decreases (Fig. 5c–f). The counter-intuitive redshift of the emission peak with decreasing temperature is strikingly different from the traditional semiconductors, where the emission peak blushifts with the decreasing temperature. This anomalous temperature-dependent band gap remains elusive and demands further investigations. The reducing FWHM with the decreasing temperature can be attributed to the weaker electron–phonon interaction at the lower temperature. For the tetragonal inclusions within the orthorhombic phase, the emission peak energy shows blueshift with the decreasing temperature (Fig. 5c), which is probably due to the increasing quantum confinement effect in the small tetragonal domains. As the temperature decreases, the size of the tetragonal inclusions decreases, leading to a stronger quantum confinement effect and thus a blueshift of emission peak. Furthermore, the FWHM of tetragonal inclusions increases with the decreasing temperature (Fig. 5e), which might be due to the size variation of the tetragonal inclusions. Therefore, the trend of the peak position and FWHM can be used to identify the phase transition points as well (Fig. 5c,e).

## Discussion

The size-dependent shift in the phase transition temperature has been extensively investigated and observed in confined systems such as nanocrystal^30^,^53^ and two-dimensional layered materials (NbSe_2_ (ref. 54) and TaS_2_ (ref. 55)). A reduction in the nanomaterial size can lead to the decrease of the structural phase transition temperature. Many competing theories have been proposed to explain this behaviour, which includes the lack of nucleation sites, internal pressure and surface energy difference between polymorphs^53^. The rough surface in our microplates can exclude the possibility of the lack of nucleation sites. The internal strain would lead to the broadening of the emission peak and thus a larger FWHM in the thinner microplates. Nevertheless, the similar or smaller FWHM in the thinner microplates (Fig. 5e,f) implies that the strain effect should not play a dominant role in our case. The thickness-dependent phase change temperature *T*_t-o_ observed here is more likely due to the surface energy difference between the polymorphs. As the thickness decreases, the surface-to-volume ratio increases, resulting in the lower transition temperature *T*_t-o_ in the thinner microplates. This explanation is consistent with the blueshift of the PL emission peak with the decreasing of the thickness due to the surface effect (Supplementary Fig. 7).

In summary, we have systematically investigated the size-dependent phase transition in individual MA lead iodide microplate using temperature-dependent PL spectroscopy, charge transport measurement and TEM studies. Our studies demonstrate that the orthorhombic-to-tetragonal phase transition temperature *T*_t-o_ decreases with the decreasing thickness of the perovskite microplates, and confirm the phase transition is a first-order solid–solid phase transition. In addition to the fundamental importance, the thickness-dependent structural phase transition has important practical implications in the fields ranging from electronics, optoelectronics to materials sciences. Our findings on the thickness- and temperature-dependent optical and electric properties can shed light on the development of electronic and optoelectronic devices not just at room temperature but also at low temperature, which would have important applications in airplanes and satellites^56^.

## Methods

### Sample preparations

A dilute PbI_2_ aqueous solution (0.1 g per 100 ml) prepared at 80 °C was cooled to room temperature, which leads to the formation of suspended PbI_2_ microplates. For the PL measurement samples, we dipped the substrates (Si substrates with 300 nm SiO_2_) with pre-fabricated markers by photolithography into the aqueous solution for a few seconds. After taking the substrate from the solution, we can find various thickness microplates by chance. For the FET samples, the 5 nm Cr/50 nm Au (Pt) electrodes with channel lengths of 8 and 40 μm were defined by photolithography and followed by thermal evaporation and lift-off. Then PbI_2_ microplates were grown onto the pre-fabricated electrodes by randomly dispersion. The prepared PbI_2_ microplates were converted into CH_3_NH_3_PbI_3_ by vapour phase intercalation. The intercalation source (MA iodide powder) was synthesized by a solution method^57^. The MA iodide source was placed at the centre of a quartz tube and the substrate with PbI_2_ microplates was placed 5–6 cm away downstream. Before conversion, the tube furnace was vacuumed and refilled with argon for at least three times to completely remove the air in the quartz tube. The conversion was conducted at a pressure of 100 mbar with 100 s.c.c.m. argon flow as carrier gas for several hours. The actual temperature is 140 °C at the MA iodide source region and 120 °C at the PbI_2_ micro-plate substrate region measured by a thermocouple probe. Finally, the tube was naturally cooled down to room temperature.

### Fabrication of graphene-contact FETs

To fabricate the graphene contact devices, graphene strips (as electrodes) was peeled on clean silicon/silicon oxide (300 nm) substrate, whereas the PbI_2_ plates and the top hexagonal boron nitride (hBN) was peeled on polymer stack PMMA/PPC (polypropylene carbonate) spun on a silicon wafer. First, the peeled PbI_2_ plate was aligned and transferred onto the graphene strips and the PMMA/PPC was dissolved by using chloroform solution. Then, the PbI_2_ plate was converted to CH_3_NH_3_PbI_3_ by using the vapour phase intercalation. Afterwards, the top BN was aligned and transferred to protect the perovskite for the following electrode fabrication processes. To make edge contact to graphene, the windows on hBN were first defined by electron-beam lithography exactly upon the graphene stripes and followed by the plasma etching to remove hBN. Afterwards, electron beam lithography was used to pattern the edge contact to graphene and followed by thermal evaporation and lift-off.

### Microscopic and optical characterizations

The thickness of the perovskite microplates was determined by tapping-mode atomic force microscopy (Vecco 5,000 system). TEM images and SAED patterns were acquired in an FEI Titan high-resolution transmission microscopy. The PL measurement was conducted under a confocal micro-Raman system (Horiba LABHR) equipped with a 600 g mm^−1^ grating in a backscattering configuration excited by an Ar ion laser (488 nm). For the low-temperature measurement, a liquid nitrogen continuous flow cryostat (Cryo Industry of America) was used to control the temperature from 77 to 300 K.

### Electrical measurements

Temperature-dependent FET device measurements were carried out in a probe station ((Lakeshore, TTP4) coupled with a precision source/measurement unit (Agilent B2902A). The scanning rate for the transport measurement is 20 V s^−1^ and the devices were pre-biased at the opposite voltage for 30 s before each measurement.

## Additional information

**How to cite this article:** Li, D. *et al*. Size-dependent phase transition in methylammonium lead iodide perovskite microplate crystals. *Nat. Commun.* 7:11330 doi: 10.1038/ncomms11330 (2016).

## Supplementary Material

Supplementary Information Supplementary Figures 1-8 and Supplementary Note 1.
