# Supplementary material for: Size-dependent phase transition in methylammonium lead iodide perovskite microplate crystals
Source: Nat Commun. 2016 Apr 21;7:11330. doi: 10.1038/ncomms11330 (PMC4844678; doi:10.1038/ncomms11330)
Supplement: Supplementary Information — Supplementary Figures 1-8 and Supplementary Note 1. [file ncomms11330-s1.pdf]

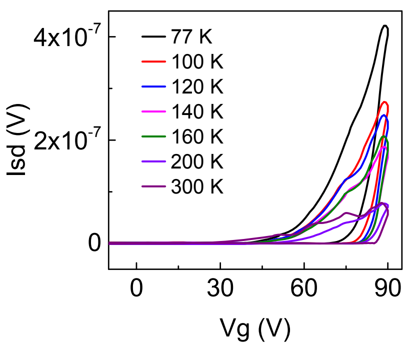

Supplementary Figure 1| The temperature dependent transfer characteristics of an individual halide perovskite microplate. The applied source-drain voltage is 20 V and the channel length is 8 μm.

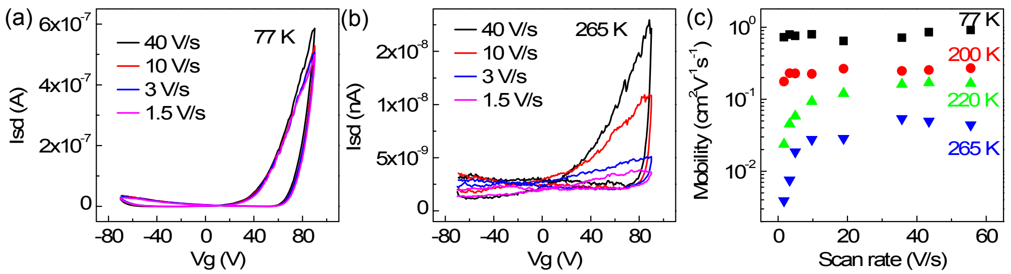

Supplementary Figure 2| The scan rate dependent transfer curve and mobility. (**a-b**) The scan rate dependent transfer curves at 77 K (**a**) and 265 K (**b**). (**c**) The scan rate dependent mobility at different temperatures.

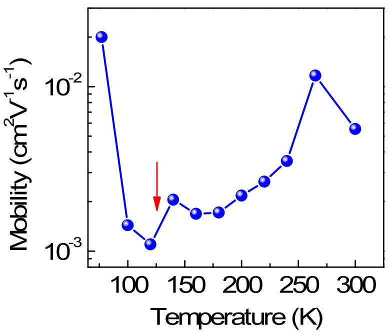

Supplementary Figure 3| The temperature dependent field-effect electron mobility of an individual halide perovskite microplate device on hBN substrate. The applied source-drain voltage is 20 V and the channel length is around 14 μm. The red arrow indicates the temperature when the orthorhombic phase to tetragonal phase transition occurs.

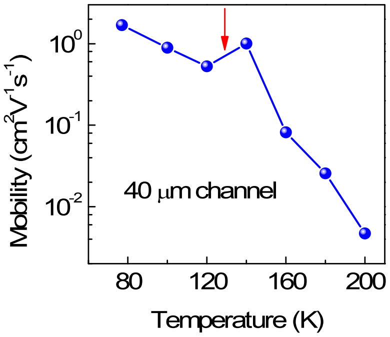

Supplementary Figure 4| The temperature dependent field-effect electron mobility of an individual halide perovskite microplate device with the channel length of 40 μm. The applied source-drain voltage is 20 V. The red arrow indicates the temperature when the orthorhombic phase to tetragonal phase transition occurs.

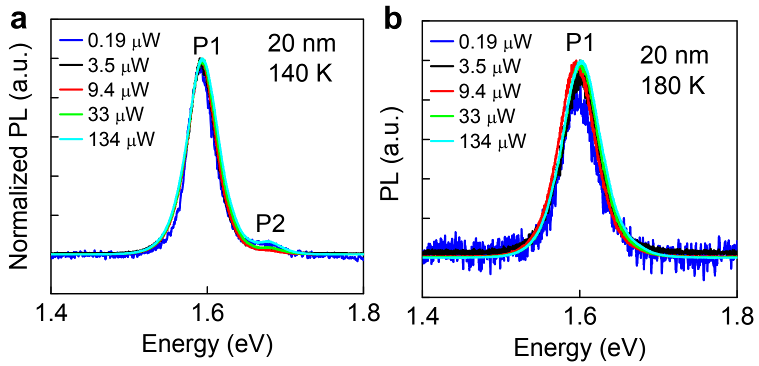

Supplementary Figure 5| The excitation power dependent photoluminescence spectra. a, **b**, The power dependent PL spectra for a 20 nm thick microplate at 140 K (**a**) and 180 K (**b**) excited by a 488 nm laser.

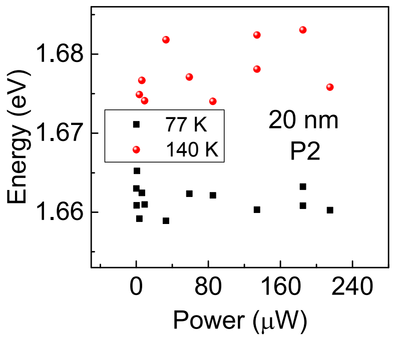

Supplementary Figure 6| The excitation power dependent emission peak energy. The excitation power dependent emission peak position of orthorhombic phase for a 20 nm perovskite microplate.

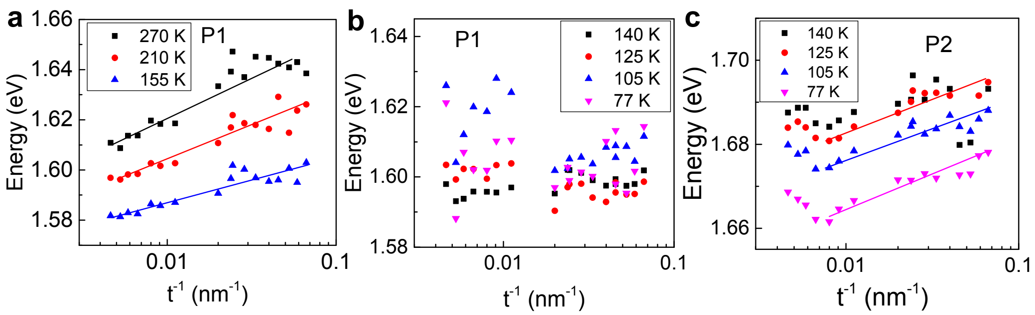

Supplementary Figure 7| The thickness dependent emission peak energy. a, The thickness dependent emission peak position of tetragonal phase at 155 K, 210 K and 270 K. **b**, The thickness dependent emission peak position of tetragonal phase inclusions within the orthorhombic phase at 77 K, 105 K, 125 K and 140 K. **c**, The thickness dependent emission peak position of the orthorhombic phase at 77 K, 105 K, 125 K and 140 K.

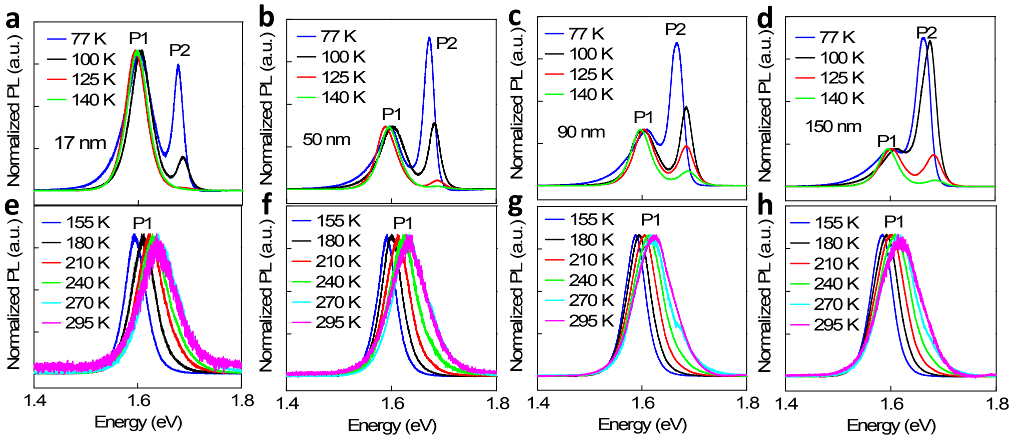

Supplementary Figure 8| The temperature dependent photoluminescence spectra. **a**, **b**, **c**, **d**, **e**, **f**, **g**, **h**, The temperature dependent PL spectra for a 17 nm (**a, e**), 50 nm (**b**, **f**), 90 nm (**c**, **g**) and 150 nm (**d**, **h**) thick halide perovskite microplates excited by a 488 nm laser with a power of 3.5 µW. All spectra are normalized by their low energy emission peak.

Supplementary Note 1: Fabrication of individual perovskite microplate field-effect transistors on the hBN substrate.

The hBN flakes were mechanically exfoliated onto 300 nm SiO_2/Si substrates. The 5 nm Cr/50 nm Au electrodes with the channel exactly on hBN were defined by electron-beam lithography followed by thermal evaporation and lift-off. Then a dry-transfer technique was used to transfer PbI_2 microplates on the pre-defined electrodes, where the PbI_2 microplates were mechanically exfoliated from a big PbI_2 crystal. Finally, the PbI_2 microplates were converted to $CH_3NH_3PbI_3$ by vapor phase intercalation as mentioned in the main text.
